# Pancreatic Cystic Neoplasms: Translating Guidelines into Clinical Practice

**DOI:** 10.3390/diagnostics13040749

**Published:** 2023-02-16

**Authors:** Sonmoon Mohapatra, Somashekar G. Krishna, Rahul Pannala

**Affiliations:** 1 Department of Gastroenterology and Hepatology, Mayo Clinic, Scottsdale, AZ 85259, USA; 2 Department of Gastroenterology and Hepatology and Nutrition, The Ohio State Wexner Medical Center, Columbus, OH 43210, USA

**Keywords:** pancreatic cystic lesions, pancreatic cancer, main duct intraductal papillary mucinous neoplasms, branched duct intraductal papillary mucinous neoplasms, mucinous cystic neoplasm, serous cystic neoplasm, guidelines

## Abstract

A combination of several factors, including the increasing use of cross-sectional imaging and an aging population, has led to pancreatic cystic lesions (PCLs) becoming the most detected incidental pancreatic lesions. Accurate diagnosis and risk stratification of PCLs is challenging. In the last decade, several evidence-based guidelines have been published addressing the diagnosis and management of PCLs. However, these guidelines cover different subsets of patients with PCLs and offer varying recommendations regarding diagnostic assessment, surveillance, and surgical resection. Further, recent studies comparing the accuracy of various guidelines have reported significant variations in the rate of missed cancer versus unnecessary surgical resections. In clinical practice, it is challenging to decide which guideline to follow specifically. This article reviews the varying recommendations of the major guidelines and results of comparative studies, provides an overview of newer modalities not included in the guidelines, and offers perspectives on translating the guidelines into clinical practice.

## 1. Introduction

Pancreatic cystic lesions (PCLs) are the most common pancreatic incidental finding on cross-sectional imaging, and their detection has increased substantially due to the widespread use of abdominal cross-sectional imaging for various indications. While the overall reported prevalence of PCLs ranges from 2.4% to 50%, the true prevalence remains unclear and varies depending on the age of the patient population and the study methodology [1,2,3,4]. Further, only a small minority of PCLs harbor dysplasia or have a potential risk of malignant transformation. Therefore, the effective clinical management of PCLs is an exercise in accurate cyst characterization and risk stratification to prevent progression to pancreatic cancer while minimizing the surveillance burden.

PCLs are classified into two major categories: mucinous and non-mucinous cysts. Mucinous neoplasms include intraductal papillary mucinous neoplasms (main duct (MD)-IPMNs, branched duct (BD)-IPMN, or mixed) and mucinous cystic neoplasms (MCNs) [5,6]. All mucinous neoplasms are considered premalignant. The most commonly encountered non-mucinous cysts include pseudocysts, serous cystic neoplasms (SCNs), solid pseudopapillary neoplasms, and cystic neuroendocrine tumors. Among these, pseudocysts have no malignant potential, whereas SCAs rarely become malignant. In clinical practice, it can be challenging to reliably differentiate between mucinous and non-mucinous PCLs. Unfortunately, there is no optimal diagnostic tool available for accurately diagnosing and risk stratifying PCLs, which can sometimes result in unwarranted surgical resection. Management decisions for asymptomatic PCLs must balance the risk of cancer with the risk of pancreatic surgery, which is associated with a mortality of 2.1% and morbidity of 30% [5].

**Figure 1:**
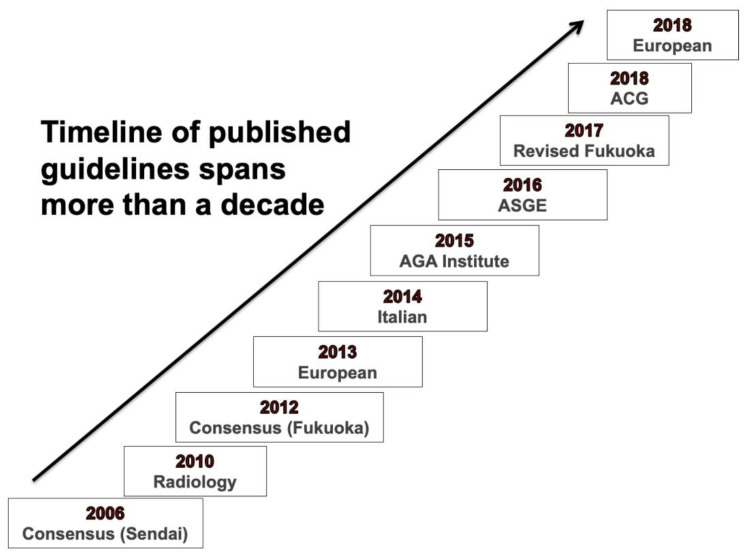
Timeline of published guidelines for the management of pancreatic cystic lesions.

In addition to the diagnostic uncertainty, there are no prospective, randomized studies that have established an ideal surveillance regimen for IPMNs. Several gastrointestinal societal guideline documents have been published in the last two decades to guide clinical decision making with regard to the diagnosis, surveillance, and treatment of PCLs [5,7,8,9,10,11,12]. The first guideline was published in 2006 [13], known as the International Consensus Guidelines or Sendai Guidelines, which was later updated in 2012 and 2017 (Fukuoka Guidelines) [11,14]. The first White paper on the management of incidental pancreatic cysts from the American College of Radiology (ACR) was published in 2010 and was revised in 2017 [7,15]. The European consensus statement on pancreatic cystic tumors was released in 2013 and revised in 2018 [12,16]. The American Gastrointestinal Association (AGA) developed practice guidelines for pancreatic cystic neoplasms in 2015 [9]., and the American Society of Gastrointestinal Endoscopy (ASGE) published their guidelines in 2016, emphasizing the role of endoscopy in the diagnosis and treatment of PCLs [10]. The American College of Gastroenterology (ACG) published its guidelines in 2018 (Figure 1) [5].

In the context of these various recommendations, decision making in clinical practice can be challenging, and clinicians are therefore unlikely to strictly adhere to a specific guideline. A comparative analysis of the various recommendations provided by the major guidelines and studies that have compared the outcomes of these guidelines can be helpful for decision making in the clinic. In recent years, a few review articles have been published comparing the societal guidelines for the management of PCLs [6,17,18,19,20]. In this review, our aim is to provide a comparative overview of the recommendations from the major guidelines, summarize the studies that have compared the clinical effectiveness of the guidelines, highlight some salient clinical questions and newer diagnostic modalities not included in the guideline recommendations, and provide our perspectives on how clinicians can translate these guidelines into their practice.

## 2. Comparative Description of Recommendations from Major guidelines

The guidelines offer varying recommendations about cross-sectional imaging for diagnosis and surveillance, indications for endoscopic ultrasound (EUS) assessment and fine needle aspiration, interpretation of cyst fluid biomarkers, thresholds for surgery, and surveillance intervals. The timeline of these guidelines spans more than a decade, and in that timeframe, our understanding of the natural history of PCLs has evolved substantially. The majority of the guidelines are focused on the management of IPMNs and MCNs. The guidelines that graded the quality of the evidence stated that it is uniformly low or very low quality, their recommendations primarily relying on the data derived from retrospective studies and expert opinions. In this section, we provide a comparative description of the recommendations from major guidelines, and a summary of these recommendations is provided in Table 1.

### 2.1. Cyst Types and Patient Characteristics

Since most precancerous PCLs encountered in clinical practice are IPMNs, these tend to be the primary focus of many of the guidelines. It is important to recognize that there is a variability in the specific cyst types and patient characteristics that each guideline encompasses. The revised 2017 Fukuoka guidelines focus on MD-IPMN and BD-IPMN and, more specifically, expand their recommendations on surgical resection criteria for BD-IPMN [11,14]. The AGA guidelines cover the management of asymptomatic PCLs and do not include other cyst types, such as solid pseudopapillary neoplasms and cystic neuroendocrine tumors [9]. The ACG and European guidelines review various cyst types in more detail and provide recommendations regarding their management [5,12]. The ACG guidelines specifically mention that their recommendations do not apply to patients with a strong family history or a genetic variant predisposing them to pancreatic cancer (PC). Other guidelines do not specifically address or exclude individuals at a higher risk of PC.

### 2.2. Preferred Imaging Modality

The Fukuoka guidelines suggest pancreatic protocol computed tomography (CT) or gadolinium-enhanced magnetic resonance imaging (MRI) with magnetic resonance cholangiopancreatography (MRCP) as the preferred imaging modalities for cyst characterization [11]. The AGA and other guidelines also propose high-quality MRIs with MRCP as the optimal imaging method owing to its higher accuracy in assessing cyst–duct communication. Moreover, the MRI/MRCP does not have a risk of radiation exposure, as compared with a CT, and is less invasive than an EUS. The European guidelines suggest considering CT for specific clinical cases, especially when the identification of calcification is important, for example, in differentiating pseudocysts associated with chronic pancreatitis from other PCL types [12]. Other clinical situations where a CT may be preferable include tumor staging if there is a suspicion of malignancy or postoperative recurrence of PC. Contrast-enhanced CT is also the imaging modality of choice for patients who are not able to undergo an MRI scan (e.g., patients with metallic implants or severe claustrophobia).

According to the ACR guidelines, MRIs and contrast-enhanced or multiphase, multiple detector CTs carry similar operating characteristics, and either can be used for diagnostic or surveillance purposes. The ACR guidelines recommend a standardized reporting of six criteria in the interpretation of cross-sectional imaging for PCLs: cyst morphology and location, cyst size, possible communication to the main pancreatic duct, presence of high-risk stigmata or worrisome features, growth on follow-up imaging, and multiplicity [15].

### 2.3. Indications for EUS and Cyst Fluid Analysis

**Figure 2:**
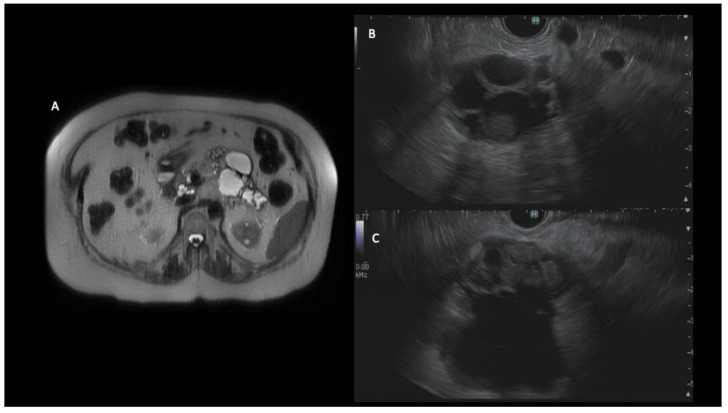
MRI of a 79-year-old female showed numerous cystic lesions with rapidly enlarging cysts in the distal body and tail of the pancreas (**A**). An endoscopic ultrasound demonstrated an approximately 50 mm cyst in the body of the pancreas associated with mural nodularity and pancreatic ductal dilation up to 20 mm (**B**,**C**). A fine-needle biopsy was obtained and showed evidence of at least high-grade dysplasia. The patient underwent distal pancreatectomy and was diagnosed with invasive moderately differentiated adenocarcinoma (T3N0M0).

The EUS is complementary to other imaging modalities in evaluating PCLs. A combination of variables such as EUS morphology, cytology, and cyst fluid biomarkers improves the diagnostic accuracy in differentiating mucinous versus non-mucinous cyst types. The guidelines have varied recommendations for EUS and EUS-guided fine needle aspiration (EUS-FNA) in the evaluation of PCLs. The Fukuoka guidelines provide a risk stratification framework for IPMNs based on ‘high-risk stigmata’ or ‘worrisome features’. [11]. High-risk stigmata include obstructive jaundice, enhancing the mural nodule ≥ 5 mm, and the main pancreatic duct (MPD) > 10 mm Figure 2. The worrisome features include cysts ≥ 3 cm, enhancing the mural nodule < 5 mm, thickened enhanced cyst walls, a MPD size of 5–9 mm, an abrupt change in the MPD caliber with distal pancreatic atrophy, lymphadenopathy, an elevated serum level of carbohydrate antigen (CA)19-9, and a rapid rate of cyst growth > 5 mm/2 years. The presence of any of the worrisome features is an indication of EUS-FNA, according to the Fukuoka guidelines. If patients with PCLs present with acute pancreatitis, the ACG and Fukuoka guidelines recommend an EUS assessment, especially if the cyst is thought to be the etiology of their symptoms [5,11].

The AGA guidelines have a higher threshold for EUS-FNA and recommend this when at least two high-risk features (size ≥ 3 cm, dilated main pancreatic duct, or the presence of associated solid components) are present [9]. The ACG guidelines recommend EUS-FNA for criteria similar to the Fukuoka guidelines but caution against solely relying on EUS-FNA for diagnosing high-grade dysplasia or malignancy in IPMNs and MCNs, given the low cellularity and low sensitivity of cyst fluid cytology [5].

The European guidelines recommend EUS-FNA for cysts when the diagnosis is unclear, and the results are expected to change the clinical management [12]. EUS-FNA should not be performed if the diagnosis is already available or there is a clear indication for surgery. The European guidelines recommend contrast harmonic enhanced EUS (CH-EUS) for the further evaluation of mural nodules [12]. CH-EUS helps to better assess the vascularity within the mural nodule, and hyperenhancement raises concerns for malignant transformation [12]. Contrast agents for EUS have not yet been approved by the Food and Drug Administration (FDA) in the United States.

The ACR guidelines recommend EUS-FNA for cysts with ‘worrisome features’ or ‘high-risk stigmata’, except for cysts ≥ 3 cm without any additional ‘worrisome features’ or ‘high-risk stigmata’, which they recommend can be followed by cross-sectional imaging [15]. The ASGE guidelines recommend EUS-FNA for PCLs > 3 cm with the presence of an epithelial nodule, dilated pancreatic duct, or suspicious mass lesion. In the absence of these features, EUS-FNA is considered optional. The ASGE guidelines suggest the administration of prophylactic antibiotics prior to performing EUS-FNA. Endoscopic retrograde cholangiopancreatography (ERCP), direct per-oral pancreatoscopy, and intraductal ultrasound may be considered for the diagnosis and characterization of suspected MD-IPMNs.

Cyst fluid analysis and biomarkers: The Fukuoka and AGA guidelines mention fluid carcinoembryonic antigen (CEA) as a marker for establishing the mucinous nature of the cyst, but they did not provide a firm recommendation on the thresholds to utilize it [9,11]. In contrast, the ACG, European, and ASGE guidelines suggest measuring the cyst fluid CEA and cytology if sufficient fluid is present [5,10,12]. The use of molecular and genetic analyses is not yet standardized and is labeled investigational in the Fukuoka guidelines [11]. The ACG, European, and ASGE guidelines suggest checking molecular markers in cases where the diagnosis is unclear, and the results would likely change the management [5,12].

### 2.4. Indications for Surgery

There are also variations in the recommended threshold criteria for the surgical resection of IPMNs. Surgical resection is recommended for patients with MD-IPMNs, MCNs, and BD-IPMNs with high-risk stigmata (obstructive jaundice, enhancing mural nodule ≥ 5 mm, and main pancreatic duct > 10 mm), according to the Fukuoka guidelines [11,14]. The Fukuoka guidelines specified the histologic subtypes of IPMNs (gastric, intestinal, pancreatobiliary, and oncocytic) and emphasized the relevance of the varying malignant potentials of these subtypes in surveillance and management decisions [11,14]. The pancreatobiliary IPMN subtype has a higher risk of malignant transformation and may be considered for surgery even without worrisome features or high-risk stigmata for malignancy.

The AGA guidelines propose a higher threshold for surgical treatment, requiring both a solid component and a dilated pancreatic duct and/or a positive cytology on EUS-FNA [9]. The ACG guidelines do not provide any recommendations regarding the criteria for resection and instead recommend multidisciplinary pancreatic group discussion for patients with IPMNs or MCNs with high-risk features, high-grade dysplasia, or pancreatic cancer on cytology [5]. Multidisciplinary discussion is recommended for all high-risk patients to review the benefits of surveillance versus surgery [5]. If a patient requires surgical resection, factors such as associated comorbidities, life expectancy, type of surgery, and the estimated morbidity and mortality associated with the surgery should be discussed by the multidisciplinary panel to assess the surgical candidacy. The ACG guidelines specifically mention referral to a tertiary care or high-volume center for all pancreatic resections, as such centers are associated with a lower rate of surgical morbidity and mortality [5].

The European guidelines recommend surgical resection for patients with characteristics similar to the Fukuoka guidelines [12]. Symptoms such as acute pancreatitis or new-onset diabetes mellitus (DM), a main PD dilation of 5–9.9 mm, increased level of serum CA 19-9, enhancing mural nodule < 5 mm, cyst size ≥ 4 cm, and cystic growth rate ≥ 5 mm/year are considered relative indications for surgical indication, according to the European guidelines [12]. The ACR recommends surgical referral for cysts showing interval growth, worrisome features, or high-risk stigmata [15].

Regarding the management of other cyst types, all of the guidelines except the European guidelines recommend consideration of the EUS or referral to a multidisciplinary group for MCN ≥ 3 cm. The 2018 European guidelines recommend surgery for MCN sizes ≥ 4 cm or MCNs that are symptomatic or have high-risk features, irrespective of their size. Surgical resection is recommended for all solid pseudopapillary neoplasms, according to the ACG and European guidelines [5,12].

### 2.5. Conduct of Surveillance

Before initiating surveillance, a thorough discussion of the risks and benefits of a surveillance program should be considered for patients with PCLs. It is important to note that certain patients may not prefer to undergo surveillance because of advanced age, associated comorbidities, or high-risk surgical candidacy. In addition, one must consider the psychosocial burden of patients undergoing routine monitoring. Surveillance is generally not recommended for patients who are unwilling to undergo surveillance or are not candidates for surgical resection.

The Fukuoka and ACG guidelines provide similar surveillance recommendations based on the cyst size, although there is some difference in the suggested intervals and surveillance modality, as detailed in Table 1 [5,11]. The AGA guidelines recommend longer intervals between surveillance examinations; all cysts < 3 cm in size and cysts with more than two high-risk features are considered together, and annual surveillance is recommended in these groups [9]. The ACR guidelines stratified their surveillance recommendations based on cyst size and patient age and recommend less frequent surveillance for patients ≥ 80 years with cyst sizes ≤ 2.5 cm or cyst sizes > 2.5 cm without any high-risk features [15]. All guidelines recommend continued surveillance after surgical resection for IPMNs but at varying intervals, as detailed in Table 1.

Regarding the cessation of surveillance, all guidelines except the AGA recommend continuing surveillance unless the patient is no longer a surgical candidate [5,11,12]. However, none of the guidelines provide specific recommendations for assessing fitness for surgery. The AGA guidelines recommend the cessation of surveillance at five years if there is no significant change in the cyst characteristics, if the patient is no longer a surgical candidate, or if there are no features of high-grade dysplasia or malignancy in the resected specimen [9]. The ACR guidelines recommend the cessation of surveillance if the cyst is stable over 10 years [15].

## 3. Comparative and Validation Studies

Several studies have assessed and compared the clinical effectiveness of the guidelines, but all studies were retrospective and performed in selected patient populations, usually among patients who underwent surgical resection for PCLs. Studies have attempted to assess the guidelines by evaluating the rate of unwarranted surgery for benign pathologies and miss rates for high-grade dysplasia or PC. Currently, there are no prospective comparative trials that assess the effectiveness of the various guideline recommendations on the management of patients with PCLs.

Singhi et al. performed a validation study assessing the accuracy of the AGA guidelines and included 225 patients who underwent EUS-FNA with a corresponding molecular analysis over 17 months. The study reported that the AGA guidelines would miss 45% of IPMNs with adenocarcinoma or high-grade dysplasia [21]. The sensitivity, specificity, positive predictive value, and negative predictive value of the AGA guidelines in identifying advanced neoplasia were 62%, 79%, 57%, and 82%, respectively, suggesting that the AGA guidelines are relatively inaccurate in identifying PCLs with advanced neoplasia [21]. However, the study population included asymptomatic and symptomatic cysts and evaluated all PCLs with EUS-FNA, which differed from the clinical decision support tool included in the AGA guidelines.

A study of 2000 randomly selected patients with presumed IPMNs retrospectively evaluated the 5-year risk of PC in Fukuoka-negative (no high-risk or worrisome features) and Fukuoka-positive (subgroups of high-risk and worrisome features) patients. The study reported a low (2–3%) 5-year risk of PC among Fukuoka-negative cysts [22]. Cysts classified as high risk were associated with a higher 5-year PC risk compared to those with worrisome features (49.7% versus 4.1%, *p* < 0.001), suggesting that the Fukuoka guidelines accurately risk stratifying PCLs into the high-risk and worrisome features category [22]. The study reported that the PC risk is highest within the first six months of feature development, especially in cysts with high-risk Fukuoka features.

Crippa et al. assessed the outcomes in individuals who met the criteria for surgery for MD-IPMN and BD-IPMN but did not have a surgical resection and noted a 96% 5-year disease-specific survival in patients over the age of >70 years, suggesting that conservative management is appropriate for this group [23]. The presence of worrisome features was associated with a better 5-year disease-specific survival compared to those with high-risk stigmata (96.2% versus 60.2%, *p* < 0.0001), suggesting that the presence of high-risk stigmata warrants surgical resection in medically fit patients [23].

Lekkerkerker et al. evaluated 115 patients who underwent pancreatic resections and compared the final pathologic outcomes with the initial indications for resection according to the Fukuoka, European, and AGA guidelines [24]. In their study, the preoperative diagnosis was correct in 72% of the patients, and surgery was considered justified in 45%. Unnecessary surgery could have been avoided in 11%, 9%, and 21% of patients if the decision to proceed with surgery was based on the Fukuoka, European, and AGA guidelines, respectively. The analysis noted that 12% of patients with high-grade dysplasia or malignancy would have been missed with the AGA guidelines, in contrast to none with the Fukuoka or European guidelines [24]. Based on the results, it was concluded that strict adherence to the current guidelines may result in unnecessary surgery in a substantial proportion of patients. Compared to the Fukuoka and European guidelines, the AGA guidelines are associated with a lower risk of leading to unnecessary surgery but are associated with a higher risk of not recommending surgery in patients with high-grade dysplasia or malignancy. The study was limited by its retrospective nature and possible selection bias due to this being a solely surgical series.

A systematic review and meta-analysis including 21 studies (15 evaluated Fukuoka, 4 evaluated AGA, and 2 evaluated both) assessed the diagnostic utility of the Fukuoka and AGA guidelines in surgically resected histologically confirmed PCLs [25]. The study showed a similar but overall unsatisfactory diagnostic accuracy of the Fukuoka and AGA guidelines, with a pooled sensitivity and specificity of 0.67 and 0.64 with the Fukuoka and 0.59 and 0.77 with the AGA guidelines for predicting advanced neoplasia [25]. However, the meta-analysis was limited by small sample sizes in multiple studies and substantial heterogeneity among the included studies.

## 4. Emerging Diagnostic Modalities Not Included in Guidelines

The accurate detection of advanced neoplasia in IPMNs remains a challenge and has been the focus of many recent studies exploring novel technologies, biomarkers, and artificial intelligence-aided imaging modalities. While a detailed description is beyond the scope of this review, it is important to note that these modalities have the potential to substantially change our approach to PCLs and that the currently available guidelines do not include recommendations specific to these modalities.

### 4.1. Biomarkers

Cyst fluid glucose is a relatively easy biomarker to measure and has been reported to have a higher sensitivity (91% versus 56%) and diagnostic accuracy (94% versus 85%) compared to fluid CEA [26]. However, this finding is relatively recent, and this marker is not included in the guidelines. There have also been multiple recent studies on the accuracy of molecular analyses of cyst fluid for cyst characterization and the presence of dysplasia within the cyst [27,28,29,30]. The presence of KRAS or GNAS mutations in the cyst fluid is reported to be highly specific for diagnosing mucinous PCLs. The molecular analysis of combined KRAS and GNAS mutations has a higher sensitivity, specificity, and diagnostic accuracy for diagnosing IPMNs and MCNs when compared to cyst fluid CEA alone [29]. A combination of KRAS/GNAS mutations and alterations in TP53/PIK3CA/PTEN has an 89% sensitivity and 100% specificity for identifying advanced neoplasia, according to the results of a recent study [27]. The current guidelines do not specify the molecular biomarkers that should be measured in cyst fluid, and both the AGA and the Fukuoka guidelines note that these are investigational, while the ACG guidelines suggest utilizing these molecular markers in selected cases.

### 4.2. Endoscopic Technology and Advanced Imaging

The clinical utility of an EUS-guided through-the-needle biopsy of the PCL wall using microbiopsy forceps (Moray Microforceps, Steris Healthcare, Dublin, Ireland) has been studied and reported to provide superior results compared to cytology alone in identifying mucinous cysts [31,32,33]. In a retrospective multicenter study, the diagnostic yield of a through-the-needle biopsy was found to be higher than cytology alone (63.2% versus 31.6%), although this was not statistically significant (*p* = 0.1) [34]. However, an EUS-guided through-the-needle biopsy was associated with a higher rate of adverse events (up to 16% in one study), the most common being intra-cystic bleeding (usually self-limiting) and mild pancreatitis [33].

Confocal laser endomicroscopy (CLE) is another modality that has been reported to be accurate in differentiating mucinous from non-mucinous PCLs and identifying high-grade dysplasia and invasive cancer in IPMNs [35,36]. In a prospective study including 144 patients with PCL ≥ 20 mm, EUS with needle-based CLE showed a higher sensitivity (98% versus 74%) and specificity (94% versus 61%) for detecting mucinous PCLs when compared with CEA and cytology [36]. Moreover, the quantification of ‘papillary epithelial width’ and ‘darkness’ under CLE are identified as the most sensitive markers in differentiating high-grade dysplasia or adenocarcinoma from low- or intermediate-grade dysplasia, but this needs further validation in larger studies [35]. A recent study has demonstrated a high intra-observer agreement and diagnostic accuracy in differentiating mucinous versus non-mucinous cysts using EUS-guided CLE [37].

### 4.3. F-18 Fluorodeoxyglucose (FDG) Positron Emission Tomography (PET) Imaging

The utility of F-18 FDG PET or F-18 PET-CT has been increasingly investigated in recent years, and the studies have shown a high diagnostic performance of F18-FDG PET for identifying malignancy in PCLs [38,39,40,41]. However, it is important to know that, although 18-FDG PET or PET/CT is noninvasive, without a risk of radiation exposure, 18-FDG is not specific to the tumor cells and has the potential to accumulate in inflammatory cells, resulting in false-positive findings. A network meta-analysis including 1018 patients from 17 direct comparison studies (8 studies used F18-FDG PET or PET/CT) showed that the positive predictive value and accuracy of F18 FDG PET in differentiating malignant from benign PCLs were significantly higher than CT or MRI in all PCLs, including IPMNs [42]. Therefore, F18-FDG PET or PET/CT may complement the Fukuoka guidelines for the preoperative management of PCLs. Additionally, F18-FDG PET or PET/CT may be considered in patients with advanced age or high-risk surgical candidates to avoid unnecessary resection of the benign PCLs.

### 4.4. Machine Learning-Based Strategies

Springer et al. developed a cyst classifier test (CompCyst) using a supervised machine learning system based on clinical features, imaging characteristics, and genetic and biochemical markers and compared the performance of CompCyst with the standard-of-care management for PCLs [43]. They reported that CompCyst was more accurate than conventional clinical tools for classifying PCLs into groups that require surgery, surveillance, or no surveillance. Application of the CompCyst test was also found to spare surgery in more than half of the patients who underwent unnecessary resection of their cysts, suggesting that these strategies have the potential to reduce patient morbidity and the economic costs associated with the current standard-of-care pancreatic cyst management practices [43].

## 5. Practice Patterns and Awareness of the Guidelines among Clinicians

With the differences in various recommendations, significant variability exists in the practice patterns and application of the guideline recommendations in real-life scenarios. However, real world data on how the guidelines are truly implemented among clinicians is limited. A survey conducted in Italy noted that most PCLs are managed by physicians in high-volume pancreas centers located in university or teaching hospitals [44]. Outside of these centers, patients are at a higher risk of treatment disparities [44]. Westerveld et al., from the United States, included 172 participants (52% academic-based endoscopists) and showed significant variability in the evaluation and management of PCLs among clinicians based on the practice setting [45]. For example, while most community practitioners (34%) would stop surveillance in a stable-sized PCL after only three years, only 9% of the academic practitioners opted to discontinue surveillance (*p* < 0.001). These data suggest that there is a substantial opportunity for improved awareness and adequate dissemination of guidelines among healthcare providers in various practice settings.

## 6. Discussion

The management of PCLs can be complex and challenging in practice given the diagnostic uncertainty, surveillance burden, patient anxiety, and adverse events associated with diagnostic interventions and surgical resection. Several guidelines are available from various societies that detail various aspects of the management of patients with PCLs. While there is substantial variability in the recommendations of the various guidelines that can limit the clinical relevance, there are several areas of concordance as well. All guidelines concur on several high-risk features (such as obstructive jaundice, enhancing mural nodule ≥ 5 mm, and main pancreatic duct diameter > 10 mm) that need prompt clinical attention. Similarly, there is a general concordance on MRIs with MRCP or contrast-enhanced CT as the preferred imaging modalities. There are several areas of discordance, including thresholds for EUS and surgery and the optimal surveillance strategies. There is also a paucity of prospective studies demonstrating a survival or cost-effectiveness benefit for these various surveillance strategies. All except the AGA guidelines recommend continued surveillance until the patient is not a surgical candidate, which imposes a substantial surveillance burden on the patients and society. Another important factor is balancing the risk of missed neoplasia with that of unnecessary surgery, and based on data from comparative studies, the AGA guidelines are associated with a higher risk of missing advanced neoplasia compared to the Fukuoka and European guidelines, though the latter are associated with a higher rate of unnecessary surgical resections for benign lesions.

In our opinion, strict adherence to any specific guideline is often difficult in clinical practice. Each guideline should be viewed within the context of the low or very low level of evidence on which they are based. In our practice, we emphasize shared decision making by explaining to the patient the lack of high-level evidence and discussing the similarities and differences among various guidelines. Before initiating surveillance or treatment decisions, we make a thorough assessment of the patient’s surgical candidacy and the associated comorbidities. Additionally, we also discuss with patients that surveillance may be associated with risks, including the need for additional interventions to follow up on changes in the cyst morphology and other incidental findings in the abdomen. We inform all patients that an overall survival benefit has not been demonstrated with any of the surveillance strategies. We also consider various other important factors that may impact the patient relative risk perception, including a family history of pancreatic cancer. In this context, we discuss with patients that the absolute risk of malignant transformation is low for incidentally detected PCLs. In clinical practice, several issues such as the ability of the individual patient to follow through with the recommended surveillance strategy and the psychological impact are also factors that can determine the strategy for an individual patient. Access to high-volume pancreatic surgery centers, high-quality endosonographers, and a multidisciplinary team for decision making are some of the important determinants for the effective management of patients with PCLs. In our practice, any patient with high-risk or worrisome features are also discussed in a multidisciplinary conference prior to any surgical intervention, and the recommendation of the group is further discussed with the patient prior to any intervention.

Several salient questions are not currently addressed in any of the current guidelines. For example, what is the best approach to assess surgical candidacy in patients with PCLs? Should there be a different surveillance strategy for individuals with a family history of PC? How should PCLs in individuals with high-risk syndromes be managed? When do we cease surveillance, especially for low-risk lesions? There is also limited data on the cost-effectiveness of the various surveillance strategies. Studies are also needed to demonstrate whether there is an overall survival benefit and/or stage shift in PC based on these surveillance strategies. An ongoing prospective, multicenter, randomized study (EA-2185, NCT04239573) was designed to compare the outcomes of low-intensity versus high-intensity surveillance, including biomarker performance, psychological burden, and cost-effectiveness [46]. The emergence of newer technologies, biomarkers, and artificial intelligence-aided strategies is also enhancing our ability to diagnose and treat PCLs more effectively. Future guidelines should attempt to address these salient questions and the role of newer technologies in the effective surveillance of patients with PCLs.

## Figures and Tables

**Table 1:** Summary of the guideline recommendations for the management of pancreatic cystic lesions *.

	AGA (2015)	Fukuoka (2017)	ACR (2017)	ACG (2018)	European (2018)
Target patient population	Asymptomatic PCLs Do not apply to solid pseudopapillary neoplasms, cystic neuroendocrine tumors, and MD-IPMN without branch duct involvement	MD-IPMNs and BD-IPMNs	Incidentally discovered asymptomatic PCLs	All newly diagnosed PCLs without a strong family history of pancreatic cancer or genetic variants known to predispose to pancreatic cancer. Included all cyst types: Neoplastic pancreatic cysts (MD-IPMNs, BD-IPMNs, MCNs, solid pseudopapillary neoplasms, cystic NETs, serous cyst adenomas) and non-neoplastic (pseudocyst)	All PCLs PCLs are classified into 4 distinct categories epithelial neoplastic (IPMNs, MCNs, serous cystadenomas, solid pseudopapillary neoplasms, serous cystadenocarcinomas, cystic NETs), epithelial nonneoplastic (retention cysts, congenital cysts, lymphoepithelial cysts), nonepithelial neoplastic (lymphangiomas, sarcomas) nonepithelial nonneoplastic (pseudocysts, parasitic cysts)
Choice of imaging modality	MRI	Pancreas protocol CT or MRI-MRCP	Contrast-enhanced MRI or pancreas-protocol CT	MRI-MRCP	MRI-MRCP
Indications of EUS and FNA	≥2 high-risk features: Size ≥ 3 cm, dilated MPD, or presence of a solid component	Any of the following present “**worrisome features**”: Clinical pancreatitis secondary to cyst, cyst ≥ 3 cm, enhancing mural nodule < 5 mm, thickened or enhancing cyst walls, MPD 5–9 mm, abrupt change in the caliber of the PD with distal pancreatic atrophy, lymphadenopathy, increased serum CA 19-9, cyst growth rate ≥ 5 mm/2 years	Presence of any of the “worrisome features” or “high-risk stigmata” with the exception of cyst ≥ 3 cm without any additional “worrisome feature” or “high-risk stigmata” that alternatively can be followed. Worrisome features: cyst ≥ 3 cm, thickened/enhancing cyst wall, nonenhancing mural nodule, MPD ≥ 7 mm. High-risk stigmata: obstructive jaundice, enhancing solid component, MPD	Any of the following present: Obstructive jaundice or acute pancreatitis secondary to the cyst, presence of mural nodule or solid component, MPD > 5 mm, change in the caliber of PD with upstream atrophy, cyst size ≥ 3 cm, increase in cyst size ≥ 3 mm/year	PCLs with clinical or radiological features of concern for malignancy
Cyst fluid analysis	Positive cytology—highest specificity in diagnosing malignancy	Cytological and molecular analysis—considered investigational—should be performed only in expert centers	Recommend cyst fluid aspiration (cyst fluid CEA and cytology) to differentiate mucinous and non-mucinous PCLs	Cyst fluid CEA may be considered to differentiate IPMN and MCN from other cyst types. Recommended to send cyst fluid cytology Molecular markers may be considered in cases with unclear diagnosis	Cyst fluid CEA combined with cytology or *KRAS/GNAS* mutation analysis recommended for better diagnostic performance
Indications of surgery	2 criteria should be met: solid component and a dilated duct and/or concerning features on EUS and FNA	Presence of “High-risk stigmata”: Obstructive jaundice with cystic lesion in the head of the pancreas, enhancing mural nodule ≥ 5 mm, MPD ≥ 10 mm, MD-IPMN, cytology suspicious or positive for malignancy	Presence of any of the “worrisome features” or “high-risk stigmata” with the exception of cyst ≥ 3 cm without any additional “worrisome feature” or “high-risk stigmata” that alternatively can be followed.	Recommend ‘multidisciplinary referral’ for following: Cytology showing high-grade dysplasia or malignancy, mural nodule, concerning features on EUS, all MD-IPMNs, solid pseudopapillary neoplasm	Absolute indications: Obstructive jaundice, presence of an enhancing mural nodule (≥5 mm) or a solid component, positive cytology or MPD ≥ 10 mm Relative indications: MPD dilatation between 5 and 9.9 mm, cystic growth rate ≥ 5 mm/year, increased level of serum CA 19.9 (>37 U/mL), symptoms (new-onset diabetes or acute pancreatitis), enhancing mural nodules (<5 mm), and/or a cyst diameter ≥ 40 mm
Surveillance interval based on cyst size 1–2 cm	MRI in 1-year	MRI or CT in 1-year	Surveillance recommendations differ based on cyst size (i.e., <1.5 cm, 1.5–2.5 cm, >2.5 cm), patient age, interval growth, and presence of risk factors.	MRI in 1-year	MRI or EUS in 6 months in conjunction with CA 19-9 and clinical evaluation
2–3 cm	MRI in 1-year	EUS in 3–6 months		MRI or EUS in 6–12 months	MRI or EUS in 6 months in conjunction with CA 19-9 and clinical evaluation
3–4 cm	MRI in 1-year	MRI or EUS in 3–6 months		MRI or EUS every 6–12 months (multidisciplinarygroup referral)	MRI or EUS in 6 months in conjunction with CA 19-9 and clinical evaluation
Discontinuation of surveillance	If no significant change in the cyst characteristics after 5 years of surveillance or if the patient no longer surgical candidate	No data to evaluate discontinuation of surveillance	Advocate 9- to 10-year follow-up for most patients, terminating at the age of 80 years.	Lifetime surveillance unless the patient is no longer surgical candidate	Lifetime surveillance unless the patient is no longer surgical candidate
Surveillance after surgery	MRI surveillance every 2 years	IPMNs—require lifetime surveillance—every 6–12 months	No comment on post-surveillance resection strategies	IPMN—every 2 years if no remnant cyst. IPMN in remnant pancreas—surveillance based on the largest IPMN MCNs without cancer—no surveillance Solid pseudopapillary neoplasm—yearly surveillance for at least 5 years SCAs, pseudocyst, other benign cysts—no surveillance	IPMN with high-grade dysplasia or MD-IPMN—every 6 months for the first 2 years, followed by yearly surveillance. IPMN with low-grade dysplasia—followed the same manner as non-resected IPMN IPMN in the remnant pancreas, no high-grade dysplasia or MD-IPMN—followed same manner as non-resected BD-IPMN

PCLs: Pancreatic cystic lesions; IPMN: Intraductal papillary mucinous neoplasms; MD-IPMN: Main duct intraductal papillary mucinous neoplasms; BD-IPMN: Branched duct intraductal papillary mucinous neoplasms; MCNs: Mucinous cystic neoplasms; NET: Neuroendocrine tumor; MRI: Magnetic resonance imaging; MRCP: Magnetic resonance cholangiopancreatography; CT: Computerized tomography; PD: Pancreatic duct; MPD: Main pancreatic duct, CEA: Carcinoembryonic antigen; CA 19-9: Carbohydrate antigen 19-9; EUS: Endoscopic ultrasound; FNA: Fine-needle aspiration. * The table does not include ASGE guidelines, as they have solely discussed the role of endoscopy in the diagnosis and treatment of PCLs.

## Abbreviations

PCLs	Pancreatic cystic lesions;
PC	Pancreatic cancer;
MD-IPMNs	Main duct intraductal papillary mucinous neoplasms;
BD-IPMNs	Branched duct intraductal papillary mucinous neoplasms;
MCN	Mucinous cystic neoplasm;
SCN	serous cystic neoplasm;
AGA	American Gastroenterological Association;
ACG	American College of Gastroenterology;
ASGE	American Society of Gastrointestinal Endoscopy;
ACR	American College of Radiology;
MRI	Magnetic resonance imaging;
MRCP	Magnetic resonance cholangiopancreatography;
CT	Computed tomography;
EUS	Endoscopic ultrasound;
CH-EUS	Contrast harmonic enhanced endoscopic ultrasound;
EUS-FNA	Endoscopic ultrasound guided fine-needle aspiration;
CEA	Carcinoembryonic antigen;
PD	Pancreatic duct;
MPD	Main pancreatic duct;
CA 19-9	Carbohydrate antigen 19-9;
CLE	Confocal laser endomicroscopy;
F-18 FDG PET	F-18 fluorodeoxyglucose positron emission tomography.
